# Spatial quantum-interference landscapes of multi-site-controlled quantum dots coupled to extended photonic cavity modes

**DOI:** 10.1038/s42005-025-02051-y

**Published:** 2025-04-11

**Authors:** Jiahui Huang, Alessio Miranda, Wei Liu, Xiang Cheng, Benjamin Dwir, Alok Rudra, Kai-Chi Chang, Eli Kapon, Chee Wei Wong

**Affiliations:** 1 Mesoscopic Optics and Quantum Electronics Laboratory, Department of Electrical and Computer Engineering, University of California, 420 Westwood Plaza, Los Angeles, CA 90095 USA; 2 Institute of Physics, École Polytechnique Fédérale de Lausanne, Lausanne, VD 1015 Switzerland; 3 Present Address: Xi’an Institute of Optics and Precision Mechanics, Chinese Academy of Science (CAS), 710119 Xi’an, China

**Keywords:** Nanophotonics and plasmonics, Single photons and quantum effects

## Abstract

A compact platform to integrate emitters in a cavity-like support is to embed quantum dots (QDs) in a photonic crystal (PhC) structure, making them promising candidates for integrated quantum photonic circuits. The emission properties of QDs can be modified by tailored photonic structures, relying on the Purcell effect or strong light-matter interactions. However, the effects of photonic states on spatial features of exciton emissions in these systems are rarely explored. Such effect is difficult to access due to random positions of self-assembled QDs in PhC structures, and the fact that quantum well excitons’ wavefunctions resemble photonic states in a conventional distributed Bragg reflector cavity system. In this work, we instead observe a spatial signature of exciton emission using site-controlled QDs embedded in PhC cavities. In particular, we observe the detuning-dependent spatial repulsion of the QD exciton emissions by polarized imaging of the micro-photoluminescence, dependent on the controlled QD’s position in a spatially extended photonic pattern. The observed effect arises due to the quantum interference between QD decay channel in a spatially-extended cavity mode. Our findings suggest that integration of site-controlled QDs in tailored photonic structures can enable spatially distributed single-photon sources and photon switches.

## Abstract (web summary)

Spatial profiles of light emission from semiconducting quantum dots can be modified by photonic cavity field patterns. By imaging exciton emissions, the authors discovered spatial repulsive signatures dependent on their positions in the cavity, revealing quantum interference effects that enable spatially distributed single-photon sources and switches.

## Introduction

Emission properties of quantum emitters can be modified through the Purcell effect by placing them into an optical microcavity. In such a scenario, the light emission rate and spatial profile can be modified according to the local density of states (LDOS) of photons of the optical cavity. Engineering the emission properties of quantum light emitters through the LDOS of the photonic environment helps to develop single-photon sources with high purity and brightness. Using high-quality (*Q*) optical cavities confining light to a small mode volume (*V*), coherent or strong light-matter interactions can be achieved such that the coupling strength (*g*) between excitons from the quantum emitter and the cavity photons exceeds the sum of the cavity and exciton decay rates. In such a scenario, the quasiparticle called exciton-polariton can be formed and its energy dispersion exhibits hybrid features of exciton and photon through the vacuum Rabi splitting (VRS). Strong coupling of a single quantum emitter with light is one of the key pathways toward demonstrating quantum gate operation and exciton-polariton manipulation with reduced decoherence^1,2^.

The subwavelength spatial features of confined photonic modes patterns (or LDOS) inherited from optical cavity parameters are intensively investigated using site-controlled pointlike dipole sources in the cavity, such as deterministically placed Ge quantum dots (QDs)^3^ or DNA origami^4^. Scanning electron beams can also be used as a point dipole source for deep subwavelength imaging of photonic modes^5^. Near-field scanning optical microscopy (NSOM) is also widely used for probing the evanescent contributions of the photonic crystal (PhC) cavity electrical field^6,7^, Not restricted to surface imaging through NSOM, high-energy resolution electron energy-loss spectroscopy can retrieve photonic modes at the dense core of the cavity for high-resolution tomography of the nanocavities^8^. The spatial distribution of exciton-polariton condensates^9–13^ in semiconductor microcavities is also studied using various potential traps, and the resulting confined exciton-polariton wavefunction is measured in momentum and real space^13–22^. The repulsive nature of polariton-exciton interactions also enables trapping exciton-polariton wavefunctions using an optically generated exciton reservoir, which gives flexibility for visualizing spatial modulation of the trapped exciton-polariton wavefunction in real space^17^. In summary, both intrinsic cavity photonic and exciton-polaritonic spatial distributions have been thoroughly studied. However, the spatial features of excitonic wavefunctions modified through the Purcell effect and strong light-matter interactions are less studied experimentally. It is because such features can be hardly distinguished from the photonic part since most studies on spatial features of exciton-polaritons are based on 2D materials or quantum wells (QWs) in distributed Bragg reflector (DBR) cavities where the excitonic states are spatially extended. Such features can also subtly depend on the exact position of the dipole source placed in a specific photonic mode pattern, which requires spatial controllability of the dipole. On the other hand, excitonic wavefunctions from QDs that are confined in sub-wavelength zones can be, in principle, distinguished from the photonic part. Additionally, semiconductor QDs have shown great potential for photonic quantum computing and quantum information technology, with the most recent advances^23–26^ including indistinguishable photon generation using QD cluster states^27–29^, highly coherent optically active QD spin qubits^30^, entanglement-based quantum key distribution (QKD)^31–34^, and time-bin entangled single-photon sources^35,36^.

In this work, four evenly separated site-controlled pyramidal InGaAs QDs are incorporated along the long axis (*x*-axis) of the *L*7 PhC membrane cavity to enable examination of the QD-cavity interactions at different locations. Such single site-controlled pyramidal QDs with promising properties such as deterministic QD nucleation and position control (<10 nm)^37,38^, narrow spectral inhomogeneity (<10 meV)^37–39^, excellent emission energy control (<5 meV)^40,41^, high symmetry with near-zero fine structure splitting (FSS)^42,43^, and absence of cavity feeding from the wetting layer or multiexcitonic background^44,45^, are the ideal platform for solid-state cavity electrodynamics (cQED) with applications in integrated quantum photonics and are essential for scaling up the technology^41,46–48^, Recent works have improved the cavity *Q*-factor and brought site-controlled QDs into intermediate^49^ and the onset of strong coupling regimes^50^. To date, however, direct measurements of spatial features of excitonic states interacting with photonic states are lacking. Based on our device, interactions of QDs with fundamental and higher-order cavity modes are resolved spatially along the *x*-axis of the *L*7 cavity by measuring the far-field photoluminescence (PL) image of the emission. We experimentally observed a spatial repulsion in the optical emission of QD excitons at small detuning ranges where the QDs and a spatially extended higher-order cavity mode are efficiently coupled. This is a spatial signature of QD exciton emission, which otherwise is not detected spectrally due to the intense cavity emission line, due to an innate quantum interference of QD decay channels in a spatially-extended cavity field. Such spatial features are only present in the *y*-polarized components of the QD excitons, which are parallel with the polarization of the extended higher-order cavity mode emission, and they are not present when interacting with the fundamental cavity mode, which is spatially confined. Additionally, the spatial repulsion is only present for the two QDs close to the center of the cavity but not in the two side QDs, suggesting that the observed spatial features subtly depend on the position of the QD in the cavity. Using the theory of quantum interference of QD decay channels in a cavity^51^, we estimate the coupling strength of the QD-cavity interaction from the observed phenomena. Our experimental demonstration of spatial superposition of excitonic and photonic states in QD-cavity interaction can be important for a deeper understanding of the spatial extent of a single QD exciton interacting with a cavity photonic mode pattern locally with contributions in quantum engineering and metrology. Based on this observed phenomenon, incorporating site-controlled QDs at prescribed locations in a photonic structure with tailored extended spatial patterns of photonic states can enable new integrated quantum photonic devices for on-chip quantum information processing.

## Results

### Concept of multi-QD devices and observed spatial features

**Fig. 1:**
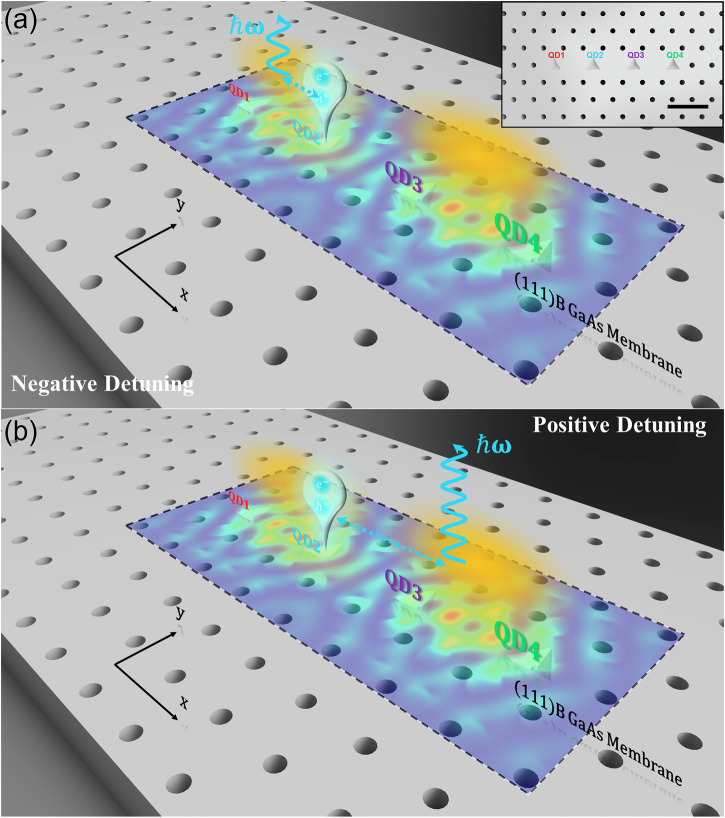
Schematic illustration of the experimental results. Schematic illustration of the spatial feature of excitonic states of QD2 at (**a**) negative and (**b**) positive detuning. Inset: Schematic illustration of the four site-controlled InGaAs QDs embedded in a *L*7 photonic crystal (PhC) cavity (PhC hole not to scale). Four QDs are arranged symmetrically with respect to the cavity center. Scale bar: 450 nm. The color pattern superimposed on the PhC cavity is referred to the calculated 1^st^ order cavity mode. The yellow clouds represent the two lobes of the spatially extended electromagnetic field of the 1^st^ order cavity mode of the *L*7 PhC cavity. The blue balloon represents the QD2 exciton. The blue dot double arrows represent the QD-cavity coupling. The wavy arrows represent the cavity-mediated QD2 exciton decay to the free space.

As shown in the inset in Fig. 1a, four site-controlled pyramidal InGaAs QDs evenly separated by 450 nm are deterministically placed along the *x*-axis in a modified *L*7 PhC cavity (hole pitch *a* = 225 nm) and are arranged symmetrically with respect to the cavity center. Figures 1a, b presents an artistic illustration of our experimental observation of the spatial superposition of the QD excitonic states in an extended cavity photonic state of the 1^st^ order cavity mode (CM1). The total electrical field intensity distribution patterns of the 1^st^ order cavity mode (CM1) calculated using the 3D finite-difference time-domain (FDTD) method are overlapped with the *L*7 cavity region in Fig. 1 (see also Fig. S2 in Supplementary Note 2 for the *x*- and *y*-polarized electric field distribution patterns of the fundamental cavity mode (CM0) and CM1). CM1 exhibits a spatially extended pattern with two spatial lobes along the *x*-axis of the *L*7 cavity, which are also artistically represented by two yellow clouds. We observe that the photo-generated QD2 exciton (blue balloon) can be coupled with the left or right lobes by controlling the detuning between exciton and CM1 energies as illustrated in Fig. 1. Such spatially distributed coupling of QD2 excitons with two lobes of CM1 can lead to photon emission (blue wavy arrows) at different locations of the *L*7 cavity.

### Optical properties

**Fig. 2:**
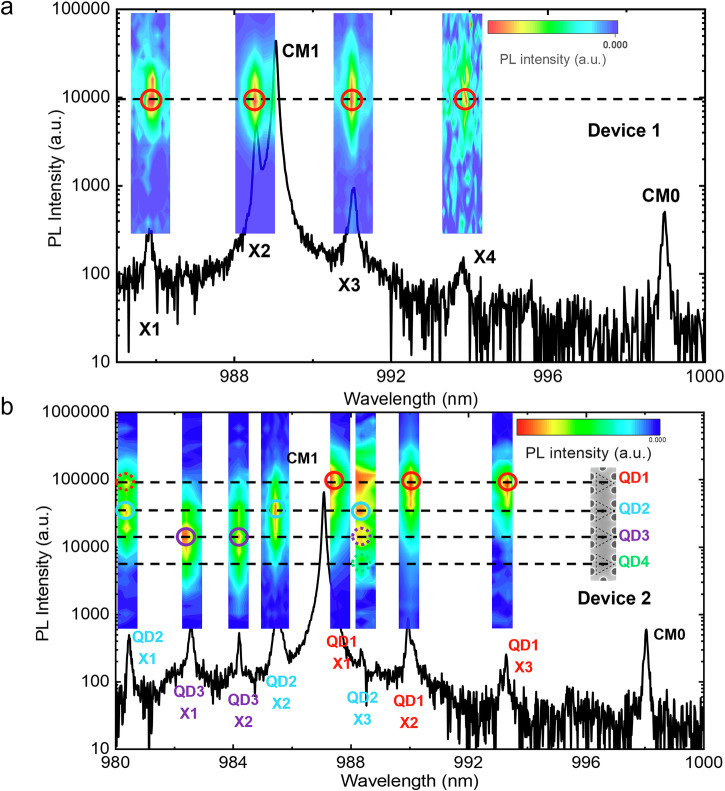
Spatial- and spectral-scanning map of four-QD exciton emissions at *T* = 10 K. **a** Device 1. **b** Device 2. The spectra at the bottom are obtained by pumping around the center of the cavity. The brightness of different QD exciton lines is shown as bars where the vertical axis corresponds to the scanning position, which is aligned with the device image on the right (for device 2).

We first excite the four QDs simultaneously using a large laser spot (≈3 *µ*m) with high power (5 *µ*W) for each device with various air hole radii. In this case, the saturated QD transitions exhibit a broadband emission. All CMs are sufficiently pumped and can be easily identified (see Fig. S3 in Supplementary Note 3). Specifically, we identify and carefully examine two 4QD-L7 devices, denoted as devices 1 and 2, with PhC hole radii *r*_1_ = 32 nm and *r*_2_ = 31 nm, respectively. These two devices exhibit good spectral overlap between QD and CM1. At a temperature T of 10 K, the *µ*PL spectra of devices 1 and 2 are measured with a small laser spot (≈1 *µ*m) at the center of the cavity as shown in the bottom spectra of Fig. 2a, b, respectively. We subsequently scan the focused laser spot across QD1 to QD4 along the *x*-axis of the *L*7 cavity. The variation in the exciton emission intensity can be used to identify the specific QDs contributing to each emission peak in the *µ*PL spectrum. The results of PL scanning are summarized as intensity bars on top of each exciton peak. For device 1, four exciton lines (X1-X4) are identified in proximity to the CM1 line and identified to be corresponding to the same QD. For device 2, eight exciton lines are observed and associated with QD1, QD2, and QD3, while QD4 is too weak to be measured. Note that some exciton emissions are contributed from different QDs simultaneously. For example, as shown in Fig. 2b, the exciton line at ≈980.46 nm is contributed by both QD1 and QD2, while the exciton line at ≈988.34 nm is contributed by QD2, QD3, and QD4 (contribution from QD4 is weak but is visible with the scale that saturates QD2 and QD3 as shown in Fig. S4 in Supplementary Note 4). Note that for the exciton line at ≈988.34 nm, the high-intensity area roughly corresponding to the QD1 position is from the saturation of CM1 intensity. For simplicity, we associate these two exciton peaks to QD2 (QD2-X1 and QD2-X3) which dominate the emission compared to the contribution from other QDs. Note that QD2-X3 can be attributed to biexciton, as it shows a 1.73 slope resulting from the linear fits to its power-dependent intensity variations as shown in Fig. S10b in supplementary Note 7. Additional QD emission features that can be attributed to charged exciton complexes or charged biexciton complexes appearing at higher power can be observed, as shown in Fig. S10a. The emission of CM0 is also observed at the longer wavelength side of the *µ*PL spectra (≈12 meV below the CM1) for both devices. The cavity *Q*-factor of device 1 is measured to be ≈12,000 for CM1 and ≈9000 for CM0. For device 2, the *Q*-factor is measured to be ≈11,000 for CM1 and ≈7000 for CM0. The observed *Q*-factor in our study is comparable to the recently reported value for single site-controlled pyramidal QDs in an *L*7 PhC cavity operating at the onset of the strong coupling regime^50^. Notably, these *Q*-factors are more than twice the previously reported values of similar devices operating in the intermediate coupling regime (*Q* ≈ 4500)^49^ or weak coupling regime (*Q* < 3500)^44,52,53^. Such improvement in cavity *Q*-factor is achieved by reducing the pyramid nominal size to approximately 200 nm, which leads to red-shifting the QD emission energy to ≈1.24 eV (≈1000 nm). At this photon energy, the reduced absorption losses from the Urbach tails of GaAs can be achieved, leading to an improved cavity *Q*-factor^50^. As shown in Fig. S5 in Supplementary Note 5, the second-order photon correlation of the cavity emission, which is tuned in between QD2-X2 and QD1-X1, exhibits photon antibunching with g^(2)^(0) ≈ 0.9. The large g^(2)^(0) value suggests that each QD emits photons individually through the cavity decay channel, and no cooperative spontaneous emission (superradiance) is observed. This lack of superradiance is likely due to the relatively large pure dephasing process, non-resonant pumping, and possible different coupling efficiency of QDs with the CM1. We notice the intense CM1 emission relative to the individual QD exciton emissions even at low power (also see Figs. S6 and S10 in Supplementary Note 6 and 7). It is likely because more than one QD contributes to the CM emission via phonon-mediated coupling. This is different than previously published works on similar structures^44,49,52,53^, for which only one QD was present in the system. Another possible reason can be due to quantum Anti-Zeno effect (AZE) that an elastic interaction between the QD and charges in the surrounding bulk material induces pure dephasing in the QD modifying the spectrum overlap between QD exciton and cavity mode and cause the strong cavity mode emission^54^. However, thoroughly investigating such an effect in our system is beyond the scope of the present work.

**Fig. 3:**
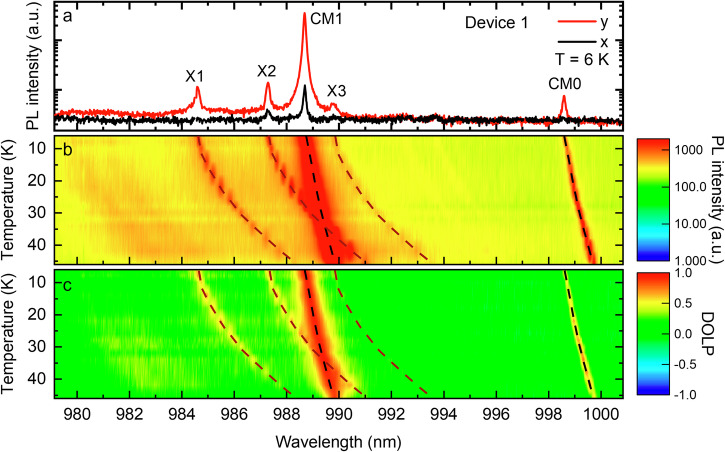
Temperature-dependent polarization-resolved microphotoluminescence of device 1. **a** PL spectrum measured at 6 K. The cavity *Q*s for CM1 and CM0 are respectively ≈12,000 and 9000. **b** PL spectra of the *y*-polarized component measured at various sample temperatures. The PL intensity is in a saturated logarithmic scale for better visibility of QD excitons. **c** DOLP measured at various sample temperatures. The dashed lines are guides to the eye of the wavelength shifts of QD exciton lines and CMs.

**Fig. 4:**
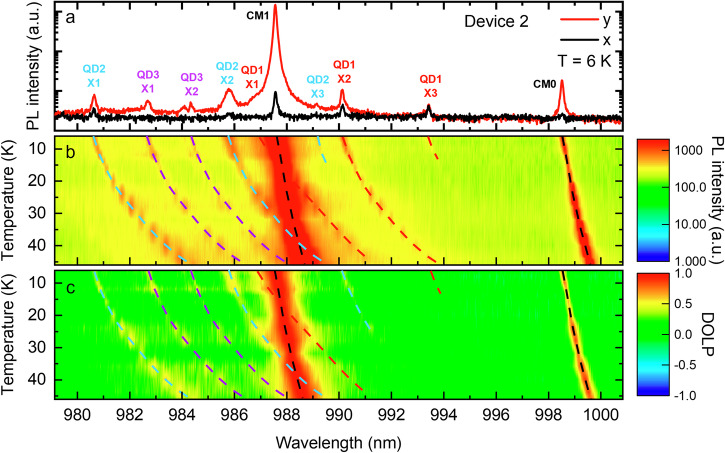
Temperature-dependent polarization-resolved microphotoluminescence of device 2. **a** PL spectrum measured at 6 K. The cavity *Q*s for CM1 and CM0 are, respectively, ≈11,000 and 7000. **b** PL spectra of the *y*-polarized component measured at various sample temperatures. The PL intensity is in a saturated logarithmic scale for better visibility of QD excitons. **c** DOLP measured at various sample temperatures. The dashed lines are guides to the eye of the wavelength shifts of QD exciton lines and CMs.

**Fig. 5:**
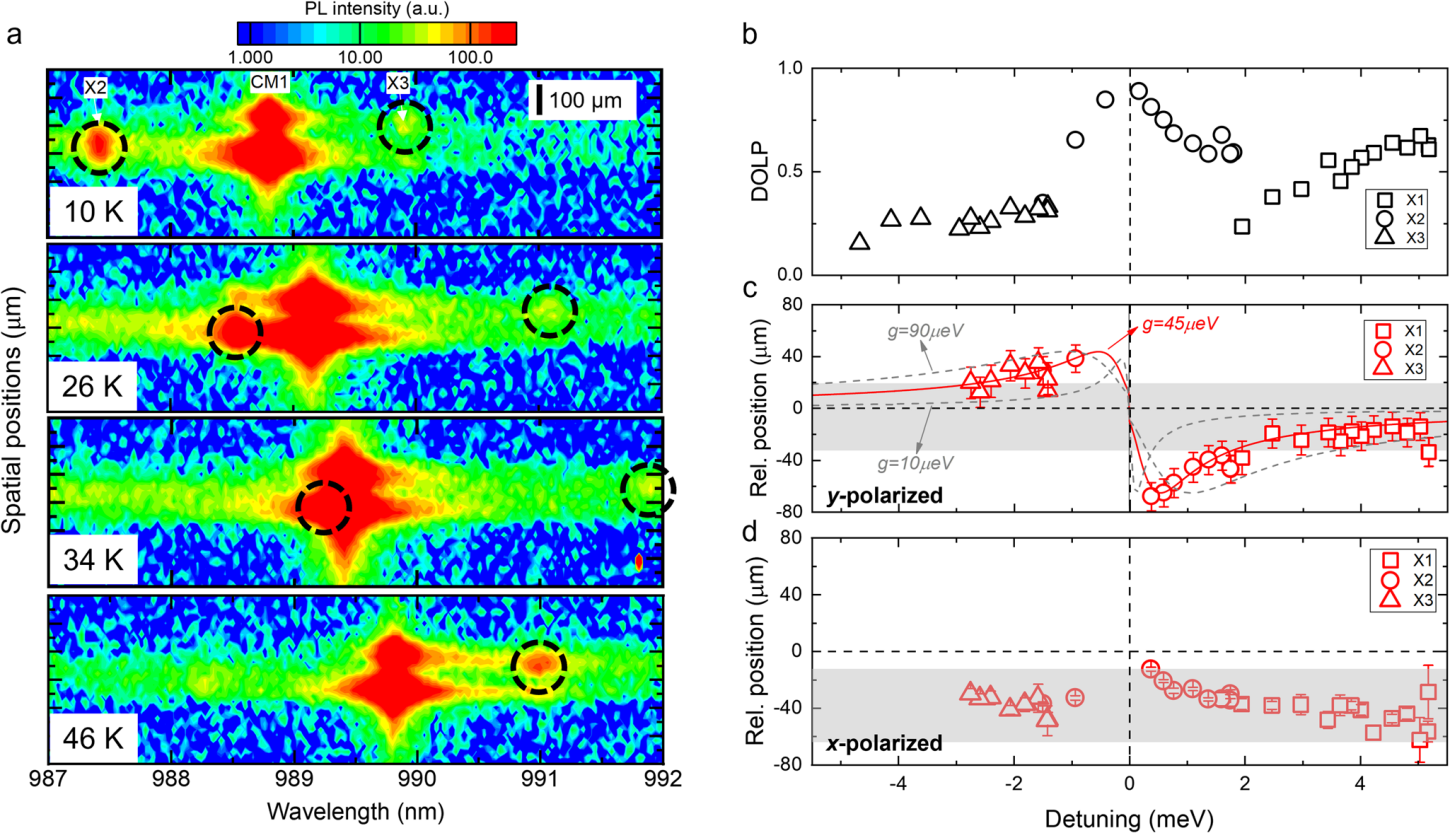
Imaging of the position-wavelength map of QD excitons and first order cavity mode (CM1) for device 1 at varying temperatures. **a** The black circles indicate X2 and X3 emission spots. The *y*-axis corresponds to the vertical axis of the CCD. The spatial resolution of the CCD is 20 μm and it is also included in the error bars. Vertical scale bar: 100 µm. Saturated logarithm color scales are used to facilitate the readibility of excitons. **b** DOLP of X1, X2, and X3 as a function of detuning. **c** *y*-polarized and **d** *x*-polarized component of the relative spatial position of X1, X2, and X3 with respect to the center of CM1 as a function of detuning. The shaded area marks approximately the span of exciton relative positions at large detuning. The red curve is the fitting using ${W}_{y1}/{W}_{y2}$ with $\kappa$ = 100 µeV, ${\gamma }_{y}$ = 0.7 µeV, ${\chi }_{21}$ = 0.9, ${\chi }_{22}$ = 0.2, and ${g}_{2}=45$ ${{{\rm{\mu eV}}}}$. The two gray cruves correspond to fitting parameters ${g}_{2}=15$ and 90 µeV.

**Fig. 6:**
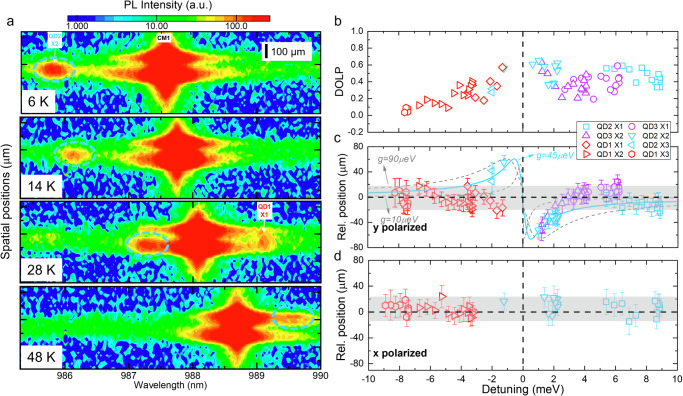
Imaging of the position-wavelength map of QD excitons and first order cavity mode (CM1) for device 2 at varying temperatures. **a** The blue circles indicate QD2 X2 emission spots. The *y*-axis corresponds to the vertical axis of the CCD. The spatial resolution of the CCD is 20 μm and it is also included in the error bars. Vertical scale bar: 100 µm. Saturated logarithm color scales are used to facilitate the readibility of excitons. **b** DOLP of excitons corresponding to QD1-3 as a function of detuning. **c** *y*-polarized and **d** *x*-polarized component of the relative spatial position of QD1-3 with respect to the center of CM1 as a function of detuning. The shaded area marks approximately the span of exciton relative positions at large detuning. The blue curve is the fitting of QD2 data using ${W}_{y21}/{W}_{y22}$ with $\kappa$ = 100 µeV, ${\gamma }_{y}$ = 0.7 µeV, ${\chi }_{21}$ = 0.9, ${\chi }_{22}$ = 0.05, and ${g}_{2}=45$ ${{{\rm{\mu eV}}}}$. The two gray cruves correspond to fitting parameters ${g}_{2}=15$ and 90 µeV.

A linear polarizer and λ/2 waveplate are used to obtain the polarization-resolved *µ*PL spectra. The degree of linear polarization (DOLP) of QD emission is determined using the expression: ${DOLP}=[({I}_{y}-{I}_{x})/({I}_{y}+{I}_{x})]$, where ${I}_{x}$ and ${I}_{y}$ are the intensity of the QD emissions in the *x*- and *y*-polarized directions. Note that CM0 and CM1 are dominant in y-polarization and exhibit large positive DOLP values due to the geometry of the *L*7 cavity. The tuning of QD exciton lines across the CM1 is achieved by adjusting sample temperatures. The temperature-dependent polarization-resolved *µ*PL and the corresponding DOLP of devices 1 and 2 are shown in Figs. 3 and 4 (see Figs. S7 and S11 for the accompanying spectral data). For device 1, as shown in Fig. 3b, excitonic transition X2 is tuned through CM1 from *T* = 6 K to 46 K, reaching a maximum DOLP of approximately 0.96 around *T* = 34 K when X2 is in resonance with CM1. Other exciton lines exhibit lower but positive DOLP values at all temperatures. For device 2, as shown in Fig. 4b, excitonic transitions QD1-X1, QD2-X2, and QD3-X2 are tuned through CM1 subsequently by adjusting temperatures from *T* = 6 K to 48 K. Large positive DOLP is observed when they are in resonance with CM1, and other exciton lines exhibit lower but positive DOLP at all temperatures (see also Figs. S7b and S11b for the accompanying DOLP spectra). We define the exciton-CM detuning as ${\delta }_{X}={E}_{X}-{E}_{{CM}}$, which represents the energy difference between exciton emission (${E}_{X}$) and CM (${E}_{{CM}}$). DOLP as a function of ${\delta }_{X}$ can serve as an indicator of the QD-cavity coupling, which is summarized in Fig. 5b for device 1 and Fig. 6b for device 2. As a result, the DOLP is positive with values >0.5 for all QDs, due to both phonon scattering and pure dephasing^49,52,53^. The co-polarization of all QDs with CM1 suggests they can be simultaneously coupled with the same CM1 for devices 1 and 2. Phonon-mediated inter-dot interaction via an off-resonant cavity mode can play a role in our system. However, revealing such phenomena requires resonantly exciting one QD that is tuned close to the cavity mode (within phonon mediation range), and subsequently measuring a transferred emission from another QD that is tuned at the other side of the cavity mode. Our current experimental setup is limited to non-resonant excitation, which is currently beyond the scope of this study.

### Spatial Repulsive Signatures of QD Exciton emissions with CM1

The exciton emissions and CM1 are then resolved spatially on the vertical axis of the CCD. To achieve this measurement, the sample is oriented such that the axis of the PL image corresponding to the *x*-axis of the *L*7 cavity is parallel to the spectrometer slit. The far-field image of the higher-order CMs, extending along the *x*-axis of the cavity, can thus be spatially mapped by a proper choice of the lens focal length for focusing the beam on the spectrometer slit. However, the position of four QDs in the studied sample cannot be effectively identified using this method as a result of the limited spatial resolution ${{{\rm{\varepsilon }}}}$ of the objective with numerical aperture (N.A.) due to the long emission wavelength $\lambda$ (${{{\rm{\varepsilon }}}}\propto \frac{\lambda }{N.A.}$) and low SNR due to the relatively weak exciton emission intensities. In Fig. 5a, the spectrally resolved diffraction-limited near field image of the spatial distribution of X2, X3, and CM1 of device 1 is shown at varying temperatures (see Figure S8 of Supplementary Note 6 for full temperature range data). Note that the vertical axis corresponds to the enlarged spatial distribution along the *x*-axis of the cavity. A saturated logarithm color scale is used to facilitate the readability of excitons. From the finite-difference time-domain (FDTD) calculated *E*-field intensity pattern, CM0 shows a single spatial lobe and CM1 exhibits two spatially extended lobes (Fig. S2). With increasing temperatures, X2 is tuned across CM1, and X3 is tuned away from CM1. The vertical positions of QD excitons are extracted by Gaussian fitting their line profile, and their relative values with respect to the center of CM1 as a function of detuning ${\delta }_{X}$ are then summarized in Fig. 5c, d for *y*- and *x*-polarized components, respectively. Interestingly, the y-polarized components of QD excitons tend to coalesce with the lower (upper) lobe of the CM1 when they are brought in resonance with the CM1 from the positive (negative) detuning side. Overall, this spatial behavior exhibits a shape of spatial repulsion within a detuning range of approximately ±3 meV, where the DOLP of QD exhibits large positive values.

In contrast, QD excitons in *x*-polarized directions do not show such spatial features as shown in Fig. 5d. Note that, in QW systems, the spatial features of the excitonic state from its superposition with the photonic state cannot be distinguished because of the extended feature of QW excitons. In our presented QD system, fortunately, the localized QD excitons show spatial coalescence with lobes of the extended photonic states at small detuning (not superimposed with photonic states at large detuning). This reveals how the subwavelength-confined excitonic states can be spatially deviated from their original state as a result of interaction with a spatially extended photonic state. Note that in the exciton-polaritonic picture, such deviated excitonic states can be regarded as exciton-polaritons with more excitonic components (lower exciton-polaritonic arm at negative detuning or upper exciton-polaritonic arm at positive detuning). Rabi spectral splitting is not resolved in this case due to the much stronger CM1 emission intensity compared to the QD exciton lines.

To further investigate the effect of QD positions, as shown in Fig. 6, a similar measurement is conducted on device 2, where QD excitons can be identified and associated with different QDs (see Fig. S12 in Supplementary Note 7 for full temperature range data). Again, CM1 exhibits upper and lower spatial lobes along the *x*-axis of the cavity, but CM0 shows only a single lobe (see Fig. S12). With increasing temperatures, QD2-X2 is tuned across the CM1 and QD1-X1 is tuned out of resonance with the CM1. QD2-X1, QD3-X1, and QD3-X2 are tuned towards the CM1, while QD2-X3, QD1-X2, and QD1-X3 are tuned away from the CM1. Vertical positions of *y*-polarized components of two center QDs (QD2 and QD3) exhibit spatial repulsion with the center of the CM1 within approximately ±3 meV detuning range, corresponding to large positive DOLP values, although QD3 is only at the positive detuning side in the measured temperature range. Interestingly, excitons from side QDs (such as QD1) do not show such phenomena. As in device 1, the spatial repulsion is not observed in the *x*-polarized component of all QD excitons, as shown in Fig. 6d.

A recent study^51^ on the DOLP proposes a theory of Fano-like^55,56^ quantum interferences between QD decay channels when it is coupled to the CM0 of an *L*3 cavity. Consider the QD placed at the antinode of the fundamental mode CM0 of a PhC cavity. The QD exciton, as a two-level system (TLS) coupled to the y-polarized CM0. The QD exciton can directly decay into x- and y-polarized unconfined free space modes (FMs), known as QD radiation mode. The exciton can also decay into the *y*-polarized FMs through the confined cavity decay channel. The phase difference between QD direct decay and cavity-mediated decay into the y-polarized FMs leads to quantum interference between the two decay pathways, which results in modified total exciton emission rate into the y-polarized FMs. Mathematically, the wavefunction of the total QD-CM system coupled to FMs can be represented by the superposition of Fock wavefunction of a single excitation in TLS, a single photon in the CM, and a single photon in the FMs. The wavefunction is governed by Schrodinger’s equation, which allows deriving the time evolution of the probability amplitude of each Fock components, resulting in a set of different equations (Eqs. S5 and S6). The total QD exciton emission rate into the y-polarized FMs (Eq. S8) as a function QD-CM detuning can be obtained by solving the eigenvalue problem (see Supplementary Note 9 for details).

In our device, inspired by ref. ^51^. and based on Eq. S8 in Supplementary Note 9, with four QDs and two lobes of CM1, for QD_j_ (*j* = 1, 2, 3, and 4), the total QD_j_ exciton decay rate into the *y*-polarized FMs considering lobe *k* (*k* = 1, 2) of CM1 is given by

1.1 $${W}_{{yjk}}=\frac{\kappa +{\gamma }_{y}}{2}-{{Re}}{\left[{\left(\frac{\kappa -{\gamma }_{y}}{2}-i{{{\rm{\delta }}}}\right)}^{2}-\left(2\left|{g}_{{jk}}\right|-i{\chi }_{{jk}}\sqrt{{\gamma }_{y}\kappa }{e}^{-i{\phi }_{{jk}}}\right)\times \left(2\left|{g}_{{jk}}\right|-i{\chi }_{{jk}}\sqrt{{\gamma }_{y}\kappa }{e}^{i{\phi }_{{jk}}}\right)\right]}^{\frac{1}{2}}$$

where $\kappa$ and ${\gamma }_{y}$ are the cavity decay rate and direct QD decay rate into *y*-polarized FMs. ${g}_{{jk}}$ is the coupling strength of QD_j_ with lobe *k*. ${\chi }_{{jk}}$ represents the spatial overlap of the direct emission patterns of QDj and field patterns of lobe *k*. ${{{\rm{\delta }}}}$ is the QD-CM detuning. ${\phi }_{{jk}}$ is the relative phase difference between decay channels, which depends on the QD positions with respect to the lobe. In this framework, observation of detuning-dependent spatial features of QD2, for instance, can be due to the dominance of ${W}_{y21}$ over ${W}_{y22}$ at the positive ${{{\rm{\delta }}}}$ and vice versa for negative ${{{\rm{\delta }}}}$. Then in the small detuning region, we could phenomenologically use ${W}_{y21}/{W}_{y22}$ to mimic the spatial feature of QD2 excitons, which is shown as the blue curve in Fig. 6c, with $\kappa$ = 100 *µ*eV, ${\gamma }_{y}$ = 0.7 *µ*eV, ${\chi }_{21}$ = 0.9, and ${\chi }_{22}$ = 0.2. For simplicity, assuming ${g}_{21}={g}_{22}={g}_{2}$, the blue curve with ${g}_{2}=45\pm 5$ *µ*eV mimic the QD2 behavior well. Similar analysis can be done on device 1, as shown as the red curve in Fig. 5c, with a coupling strength of 45 ± 5 *µ*eV. Such coupling strength aligns well with similar multi-site-controlled QD systems^57^. The coupling strength can be related to the oscillator strength $f$ and the mode volume $V$ by $g=\sqrt{\pi {e}^{2}f/4\pi \epsilon {m}_{0}V}$, where $\epsilon$ and ${m}_{0}$ are the permittivity of the cavity material (GaAs) and free electron mass, respectively. With a typical InGaAs QD oscillator strength of ~12^58^ and an estimated mode volume of ~3.42 × 10^-20^ m^3^, the coupling strength is ~ 116 *µ*eV, which is about twofold higher than measured ${g}_{2}$ likely due to misalignment of QD2 with the antinode of cavity field. The slight surpassing of ${g}_{2}$ over $\kappa /4$ suggests that our system operates at the onset of the strong coupling regime, with a small expected Rabi splitting $\varDelta E\approx 2\sqrt{{g}^{2}-\frac{{\kappa }^{2}}{16}}=37$ *µ*eV, though the experimental observation of spectral anti-crossing can be disguised by dephasing and strong CM1 emission and limited by spectrometer resolution. It is interesting to note that QD2 and QD3, which are symmetric with respect to the center of the cavity, exhibit the same spatial shape as shown in Fig. 6c. It implies the existence of the symmetry breaking of two spatial lobes of CM1, possibly due to the fabrication disorder of the PhC membrane or perturbation induced by the pyramid. This leads to additional strongly confined localized modes of CM1 spatial pattern^59,60^, an additional contribution to the above framework that needs to be further investigated. Note that, as shown in Fig. 6c, the spatial repulsive feature of QD3 (purple data) shows a slightly narrower width compared to QD2 (blue data) at the positive detuning. Therefore, it can exhibit slightly lower coupling strength, based on the trend of ${W}_{y21}/{W}_{y22}$ curves with different $g$ values as shown in Fig. S14 in Supplementary Note 7. As shown in Fig. 6c, the coupling strength for QD1 (red data) is difficult to extract because of (1) the absence of the spatial repulsive feature within the measured detuning range and (2) the low SNR of QD1 emission at the smaller detuning range. Also, QD4 is weak. Therefore, in this device, coupling strength for QD1 and QD4 is difficult to access using the above method. A thorough study of the coupling strength for QD1 and QD4 requires a better device where they emit with improved SNR or a statistical study of many similar devices. To further confirm these observations solely occurring with CM1, we extend our measurement to device 3 (PhC hole radii *r*_*3*_ = 36 nm), where we investigate the interaction of QDs with CM0 (see Supplementary Note 8). Unlike CM1, CM0 is not spatially extended and shows a single lobe. Excitons from one of the central QDs (QD3-X2 and QD3-X3) are tuned away from CM0 with increasing temperature. No spatial repulsion is observed within a negative detuning range of 4 meV. It implies that the observed spatial repulsion is correlated with the extended feature of CM1.

### Proposed spatially distributed single-photon sources and switches

**Fig. 7:**
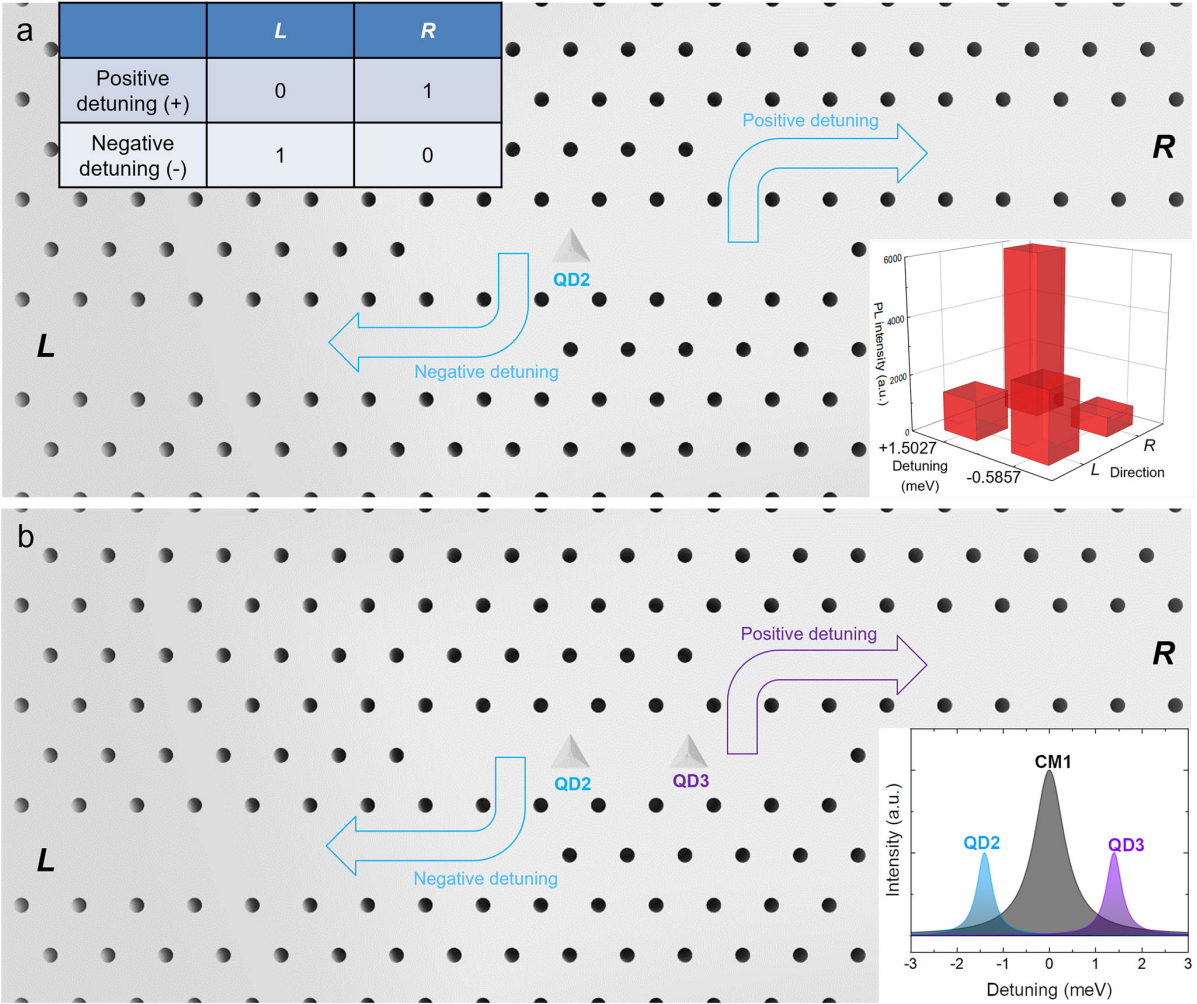
Schematic of a proposed spatially distributed single-photon source. **a** *L*7 photonic crystal (PhC) cavity with embedded QD2 evanescently coupled with two PhC waveguides denoted by *L* and *R*. Inset: logical operation of the single-photon source based on cavity detuning and measured output states in the logical bases retrieved from the PL measurements. **b** *L*7 PhC cavity with embedded QD2 and QD3 evanescently coupled with two PhC waveguides denoted by *L* and *R*. Inset: CM1 energy is tuned between QD2 and QD3, enabling a negatively-detuned QD2 exciton and a positively-detuned QD3 exciton within ± 3 meV.

Based on our experimental observation, we propose the concept of a functional spatially distributed single-photon source using site-controlled InGaAs QDs embedded in an *L*7 PhC cavity, evanescently coupled with two waveguides (WGs) along the *x*-axis as shown in Fig. 7. The two side WGs are terminated close to two lobes of the CM1 to facilitate single-photon extraction. As shown in Fig. 7a, the detuning of excitons with CM1 can be controlled for QD2 to allow single-photon propagation either to the left (negative detuning) or right (positive detuning) using evanescent field coupling. Such operation can also be expressed in the logical bases, as shown in the upper left inset of Fig. 7a. With positive detuning, the output state will be |0_L_1_R_>; with negative detuning, the output state is |1_L_0_R_>. Here we define the positive detuning as logic 0 and negative detuning as logic 1, and similarly define left-propagating photon as 0 and right-propagating photon as 1. To construct a truth table for the outputs, we retrieve the PL intensity of exciton emission when it is tuned close to CM1 from prior spatially and spectrally resolved PL measurements. Specifically, the positive and negative detuning values used are +1.5027 meV and −0.5857 meV, respectively, and the resulting truth table is shown in the lower right inset of Fig. 7a. We evaluate the output logic using the expression: $F=\frac{1}{2}{Tr}\left(\frac{{M}_{\exp }{{M}_{{ideal}}}^{T}}{{{{M}_{{ideal}}M}_{{ideal}}}^{T}}\right)$, which calculates the fidelity between the measured truth table ${M}_{\exp }$ and the ideal truth table ${M}_{{ideal}}$, and obtain an estimated fidelity of 70.76 ± 1.87% for the output states^61^. Such logic operation is suitable for building single-photon sources capable of switching operations that control photons propagating along different directions. Particularly, using optical pumping from remote quantum dots^62–64^ and dynamic Stark-based ultrafast control of detuning^65^, it can open doors for possible low-photon-number all-optical switching^66–70^, which are crucial components in linear optical and photonic quantum computers.

Based on the symmetry breaking of the CM1 lobes, Fig. 7b shows an *L*7 PhC cavity with two central QDs (QD2 and QD3) simultaneously embedded. When the CM1 emission energy falls between the QD2 and QD3 excitons, single photons from QD2 and QD3 (with different colors) can propagate to the *L* and *R* directions independently. Such behavior facilitates a tunable single-photon source for path encoding. By tuning CM1 to the middle of QD2 and QD3 (lower right inset of Fig. 7b), the single photons can be emitted through the left or right waveguides, encoded as |L> or |R>. Due to the different energies of the QD2 and QD3 emissions (ω_2_ and ω_3_), the resulting state can be expressed as |1_ω2_, _L_> or |1_ω3_, _R_>. Such a two-color tunable single-photon source behaves as a photon switch with different path encodings, which provides possibilities for spatially multiplexed quantum communications^71–73^, quantum Boson sampling^74–77^, and programmable information processing^78–85^.

## Conclusions

In summary, we report a direct experimental demonstration of the superposition of subwavelength-confined QD excitonic states from systems of four site-controlled QDs coupled to a spatially extended photonic state from a higher-order photonic mode of an *L*7 PhC cavity by wavelength-position mapping of QD-cavity interactions at varied detuning. It is observed that the QD excitonic states close to the cavity center coalesce with lobes of CM1, and such coalescence forms a spatial repulsion as a function of QD-cavity detuning. This spatial feature is a signature of the QD excitonic state and arises from the unique quantum interference between QD direct radiation modes and QD decay through two spatially separated lobes of a extended photonic cavity mode pattern. Furthermore, the symmetry breaking of two lobes of CM1 is observed, and its origin can be further investigated through NSOM or high-energy resolution electron energy-loss spectroscopy. The observed phenomena can be important to understand the spatial features of QD excitonic states from a tailored photonic environment during their interactions, with applications in spatial control of single-photon transport to remote positions using detuning, extracted by waveguides for practical photonic logic operations. Using site-controlled QD systems is necessary because the observed phenomenon is dependent on the QD position. Furthermore, based on the observed phenomena and the proposed functional all-optical switching, it paves the way towards developing programmable quantum photonic processors^78–85^ for scalable quantum information processing, engineering, and metrology.

## Methods

### Sample description and fabrication

Four site-controlled pyramidal QDs are fabricated using metalorganic vapor-phase epitaxy (MOVPE) growth of In_*x*_Ga_1-*x*_As/GaAs (*x* = 0.25) on a (111)B-oriented GaAs substrate with electron-beam lithography (EBL) inverted pyramidal pits pattern with a triangular lattice of 450 nm pitch. The precision achieved with EBL is within ±10 nm^86,87^. The resulting QDs are lens-shaped and grow at the apex of highly symmetric inverted pyramids, exhibiting well-defined (111)A Gallium-terminated facets. The nominal pyramid size is approximately 180 nm. Such a QD growing process does not involve the formation of 2D wetting layers. Instead, InGaAs/GaAs quantum wires (QWRs) are formed on the three wedges of the inverted pyramid during growth under some circumstances.

Photonic crystal structures with membrane thickness *t* ≈ 265 nm and lattice constant *a* ≈ 225 nm are lithographically written on top of the QD pattern, and all the surrounding QDs are etched away except the four QDs in the *L*7 cavity. To enhance the cavity *Q*-factor, the first three side holes on the left and right of the *L*7 cavity are shifted outwards by 0.23*a*, 0.15*a*, and 0.048*a*. A series of identical 4QD-*L*7 devices is fabricated where the air hole radii (*r*) of the photonic crystal is scanned from 30 nm to 42 nm in steps of 1 nm to facilitate the fundamental or 1^st^ order cavity mode falling in the spectral inhomogeneity of 4 QDs.

We do not have bare QDs in the batch of samples for this study. However, the optical and symmetry properties of bare site-controlled pyramidal InGaAs QD can be inferred from refs. ^88,89^. The high symmetry of such QD (${C}_{3v}$) leads to high degree of polarization isotropy of neutral exciton in the xy plane and low FSS in the range of 10–30 μeV depending on the Indium composition^90^. Our previous study using similar pyramidal InGaAs QD shows a 23 μeV FSS of the neutral exciton^49^.

### Photoluminescence measurement

The schematic of the microphotoluminescence (*µ*PL) setup is shown in Fig. S1 in Supplementary Note 1. A 900 nm laser is focused on the sample with a laser spot size of ≈1 *µ*m using a microscope objective with 100× magnification, 0.7 numerical aperture (N.A.), and 10 mm working distance. The position of the focused laser spot on the sample surface is viewed using an imaging camera. The sample is placed in a helium-flow microscopy cryostat (Janis ST-500). The relative position of the focused laser spot on the sample surface can be controlled with high-precision (30 nm) piezo linear actuators. The emission is collected by the same objective and imaged on the slit of a 1-meter-long spectrometer (Horiba 1000 M) equipped with a 2D liquid nitrogen-cooled charge-coupled device (CCD) with a spectral resolution of 40 *µ*eV. The optical spectra are then obtained by integrating the vertical axis of the PL image. The residual laser light is filtered with a long-pass (LP) optical filter. A linear polarizer and *λ*/2 waveplate are placed in front of the spectrometer for polarization-resolved *µ*PL measurement. For the second-order photon correlation measurements, the *µ*PL spectra cleaned by tunable long and short pass filters are coupled to a 50:50 single-mode fiber beam splitter. Superconducting nanowire single-photon detectors (SNSPDs, PhotonSpot) and a time-correlated single-photon counting unit (PicoHarp 300) are used for building the histograms^91^.

### Supplementary Information

Schematics of the experimental setup, additional experimental data analysis, and theoretical derivation can be found in the accompanying Supplementary Information.

## Supplementary information

Supplementary Information

## Data Availability

The data that support the findings of this study are available from the corresponding authors upon reasonable request.
