# Supplementary material for: Spatial quantum-interference landscapes of multi-site-controlled quantum dots coupled to extended photonic cavity modes
Source: Commun Phys. 2025 Apr 11;8(1):152. doi: 10.1038/s42005-025-02051-y (PMC11991910; doi:10.1038/s42005-025-02051-y)
Supplement: Supplementary file 1 — Supplementary Information [file 42005_2025_2051_MOESM1_ESM.pdf]

Supplementary Information

Spatial quantum-interference landscapes of multi-site-controlled quantum dots coupled to extended photonic cavity modes

Jiahui Huang[1, *, †], Alessio Miranda[2], Wei Liu[1, *], Xiang Cheng[1], Benjamin Dwir[2], Alok Rudra[2], Kai-Chi Chang[1], Eli Kapon[2], and Chee Wei Wong[1,*]

[1] Mesoscopic Optics and Quantum Electronics Laboratory, Department of Electrical and Computer Engineering, University of California, Los Angeles, 420 Westwood Plaza, CA 90095, USA

[2] Institute of Physics, École Polytechnique Fédérale de Lausanne, Lausanne, VD 1015, Switzerland

†Current address: Xi'an Institute of Optics and Precision Mechanics, Chinese Academy of Science (CAS), 710119, Xi'an, China

* Correspondence : jiahuihuang@ucla.edu; weiliu01@lbl.gov; cheewei.wong@ucla.edu

Supplementary Note 1: Microphotoluminescence setup

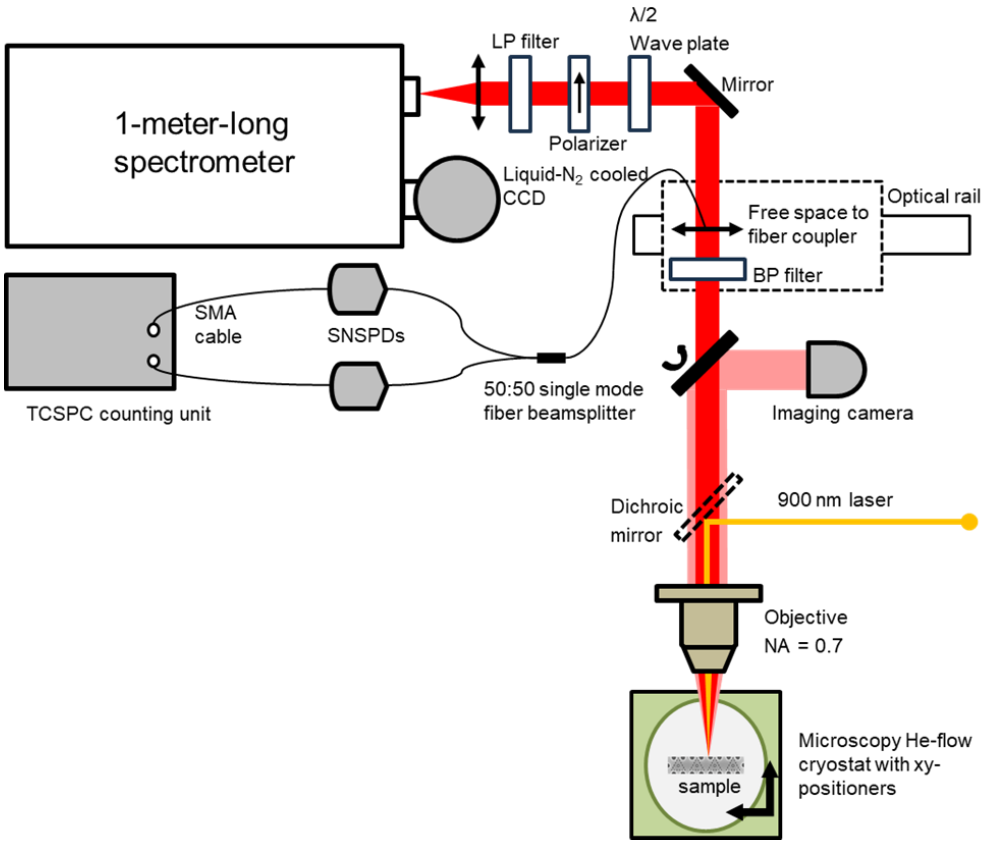

Figure S1 | Schematics of the microphotoluminescence setup. The laser path is in orange and the PL path is in red. The white light path for imaging is in pink.

Supplementary Note 2: 3D FDTD simulation of the electric field distribution of a *L*7 PhC cavity

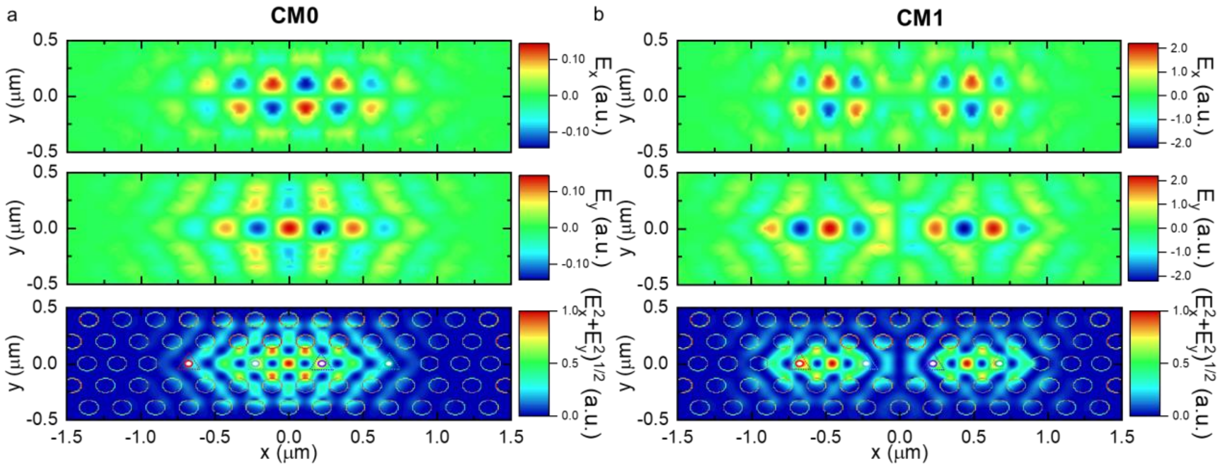

Figure S2 | 3D FDTD simulation of the spatial pattern of electric field E_x, E_y, and the total intensity $(E_x^2 + E_y^2)^{1/2}$ of the fundamental cavity mode (CM0) and 1st order cavity mode (CM1) of the *L*7 PhC cavity. Parameters: hole pitch *a* = 225 nm, membrane thickness *t* = 250 nm, air hole radius *r* = 31 nm.

Supplementary Notes 3: PL measurement of all fabricated 4QD-L7 cavity devices using high power excitation

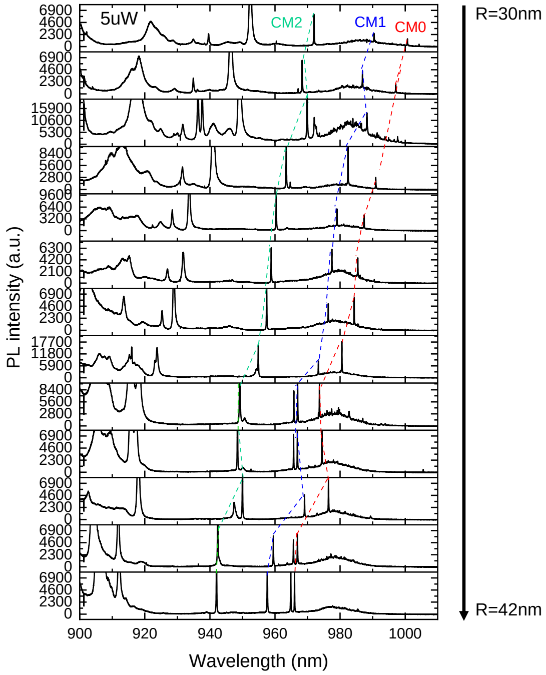

Figure S3 | PL spectra of all 4QD-*L*7 cavity devices using a large laser spot exciting all four QDs with high pump power (λ_{exc} = 785 nm, P_{exc} = 5 μW). The air hole radius of the PhC changes from *r* = 30 nm to 42 nm from top to bottom. Shift of CM0, CM1, and CM2 emission are marked by red, blue, and green dash lines. T = 10 K.

Figure S3 shows the PL spectra of 13 4QD-*L*7 cavity devices using a large laser spot (λ_{exc} = 785 nm, P_{exc} = 5 μW) covering all four QDs with at T = 10 K. In this case, QD transitions are saturated and show broadband emissions. All CMs are sufficiently pumped and can be easily identified. Shift of CM0, CM1, and CM2 emissions are marked by red, blue, and green dash lines. Quantum wire (QWR) emissions can also be observed at shorter wavelengths.

The cavity mode should exhibit continuous change if the actual steps in hole radius is continuous. But in the actual sample, fabrication disorder and errors can lead to discontinuous change in hole radius and thus the discontinuity in the cavity mode energy change or likely mode splitting, for example mode splitting of CM0 for R = 41 and 42 nm.

Supplementary Notes 4 : Results of PL scanning of device 2 at a different intensity scale

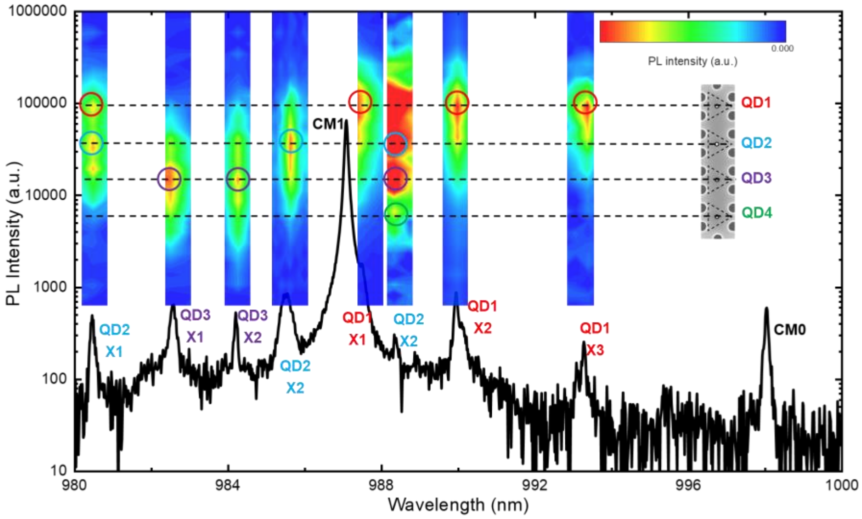

Figure S4 | Verification duplicate of PL scanning of device 2 as shown in Figure 2b. Here the intensity scale of the bar on top of QD2-X2 is set to saturate the QD2 emission for better visibility of contributions from QD4.

Supplementary Notes 5: Photon correlation measurements of QD-cavity devices

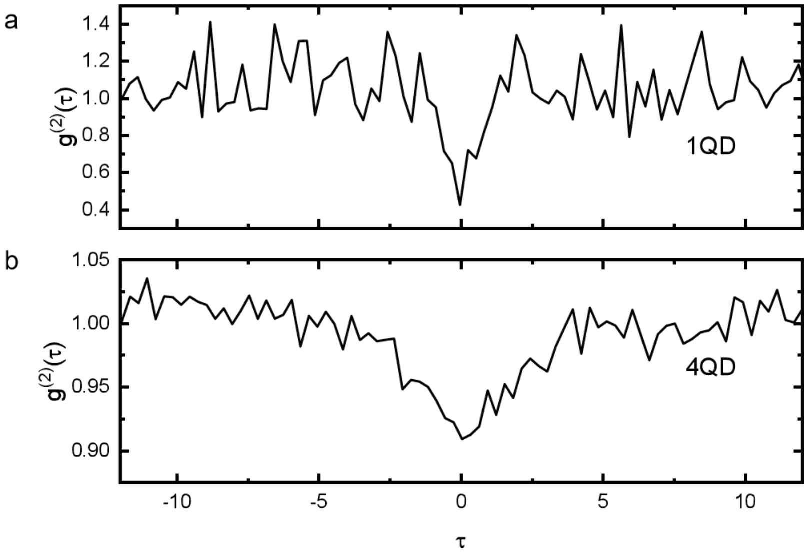

Figure S5 | Second-order correlation measurements. a, 1QD and **b,** 4QD in a $L7$ PhC cavity.

For 1QD case, QD is deterministically placed at the center of a $L7$ PhC cavity and a QD exciton line is tuned in resonance with the CM0. A narrow bandpass filter is used to select only CM0 for feeding the HBT setup. The observed antibunching with $g^{(2)}(0) \approx 0.4 < 0.5$, as illustrated in Figure S5a, supports that single

QD coupled with CM0 is at the single-photon level. The non-zero value of $g^{(2)}(0)$ is likely due to finite time response of the system and non-resonance pumping induced dephasing.

For 4QD case, device 2 in the main text is measured. CM1 is tuned between QD2-X2 and QD1-X1 with detuning $E_{QD2-X2} - E_{CM1} \approx 1.39\, meV$ and $E_{QD1-X1} - E_{CM1} \approx -1.15\, meV$. A narrow bandpass filter is used to select only CM1 for feeding the HBT setup. A weak antibunching with $g^{(2)}(0) \approx 0.9$ is observed, as illustrated in Figure S5b. It can be the result of uncorrelated single-photons emitted through the CM1 decay channel by each QD and no photon bunching is observed suggests that no cooperative or superradiance emission occurs in our system likely due to large dephasing process because of non-resonant pumping. Different coupling efficiency of QDs with CM1 can lead to cavity decay channel dominated by a specific QD so a measurement of $g^{(2)}(0)$ as a function of detuning with fine steps would be needed to observe the possible photon bunching. Note that superradiance is is more stringent on the detuning condition than co-polarization [1],[2]. On the other hand, the requirement for multi-QDs co-polarization with cavity is less demanding in a relatively large detuning, thanks to the phonon-assisted coupling , e.g. a few meV corresponding to the phonon energy. In addition, co-polarization of QDs with the cavity mode only indicates each QD coupled to the same cavity mode individually, but does not guarantee inter-QD talking or the formation of collective emission states. In Figure S5, the weaker fluctuation of 4QD case than 1QD case which is due to much higher photon counts in 4QD case during the measurement.

Supplementary Notes 6: Additional experimental results on device 1

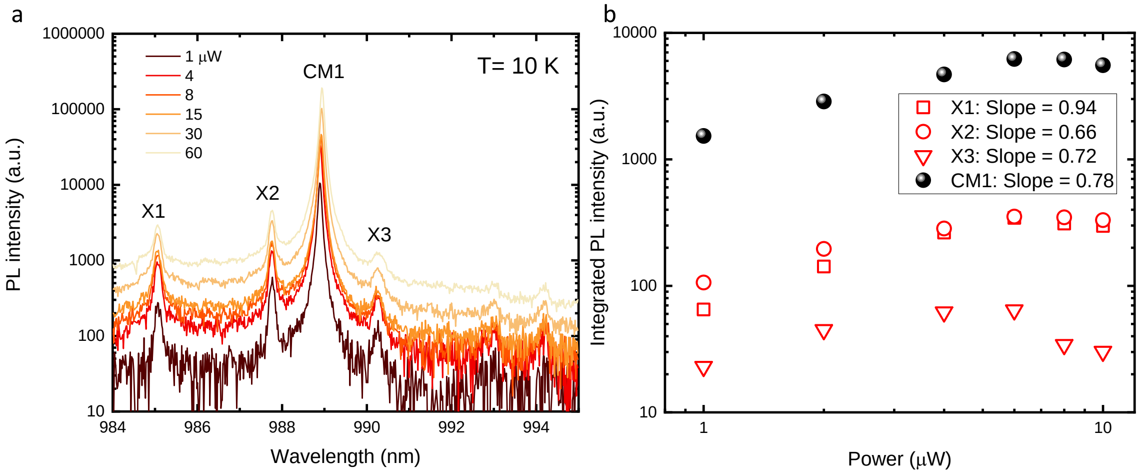

Figure S6 | Excitation power dependent PL measurement of device 1. a, Power-dependent PL spectrum of device 1. T = 10 K. **b,** Integrated PL intensity of QD exciton as a function of pump power. Linear fits to the data leads to slopes that are specified in the legend. λ_{exc} = 900 nm.

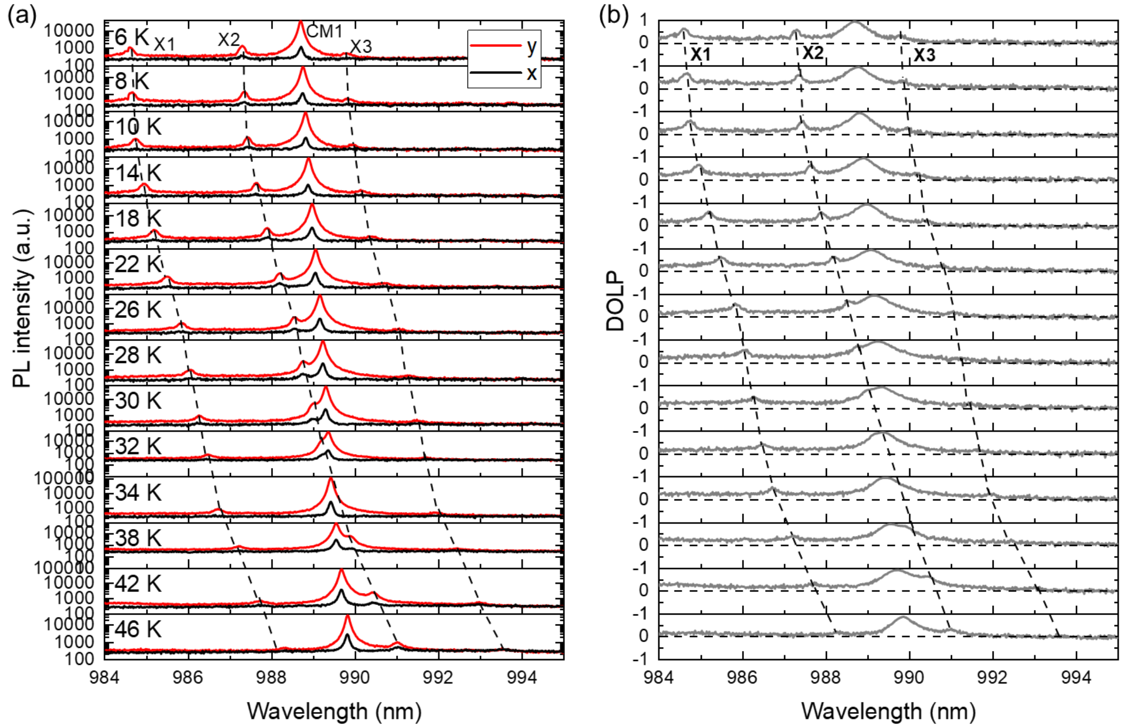

Figure S7 | Comprehensive temperature-dependent measurement data of device 1. a, Temperature-dependent polarization-resolved μPL of device 1. **b,** Temperature-dependent DOLP of device 1. Temperature Temperature varies from 6 K to 46 K. λ_{exc} = 900 nm.

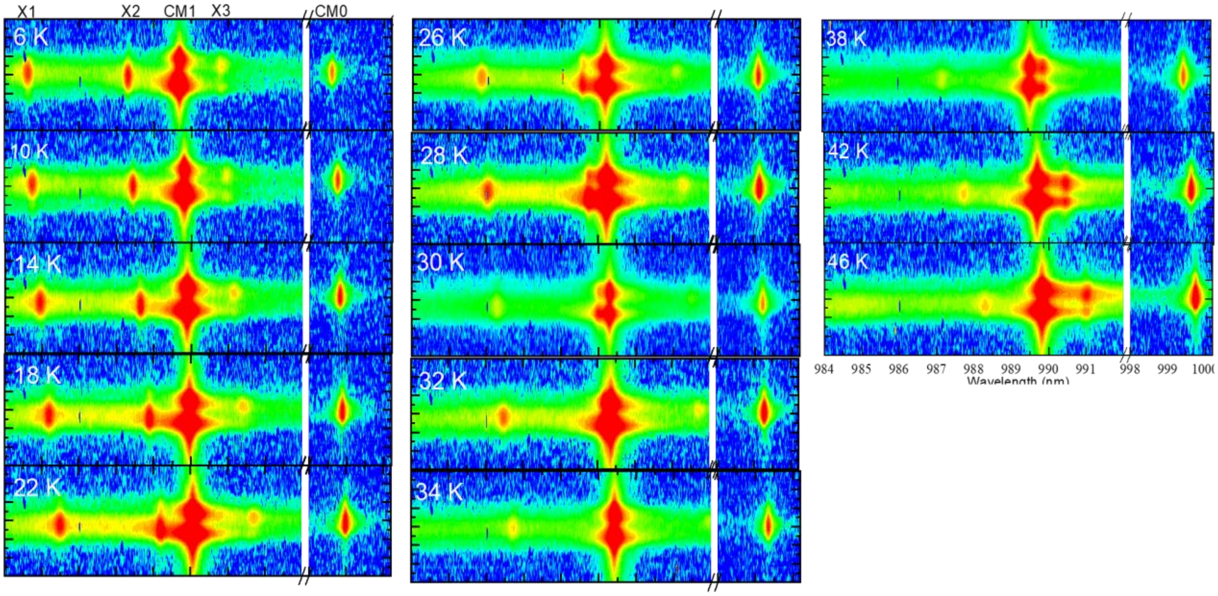

Figure S8 | CCD image of the temperature dependent position-wavelength map of QD excitons and CM1 for device 1. Temperature varies from 6 K to 46 K. λ_{exc} = 900 nm.

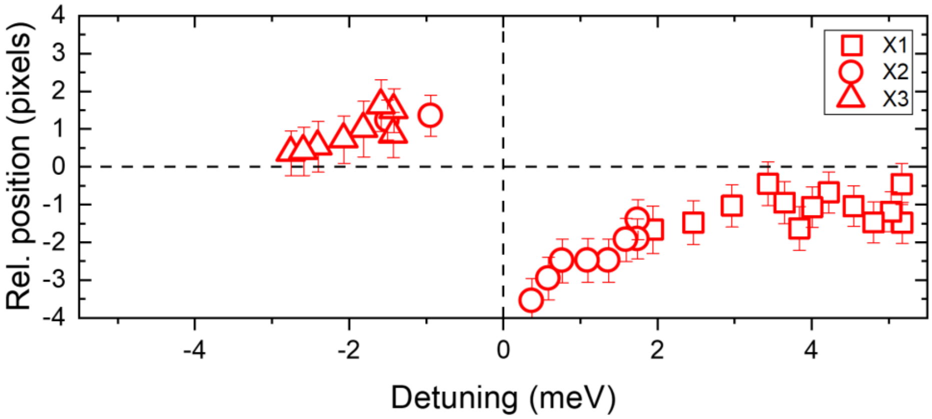

Figure S9 | The relative spatial position of QD excitons X1, X2, and X3 with respective to the center of CM1 as a function of detuning. Mixed polarization is examined here.

Polarization-resolved PL spectra (including DOLP) and position-wavelength mapping of device 1 for the whole temperature range are shown in Figures S7 and S8. The relative spatial position of X1, X2, and X3 with polarization not resolved are summarized in Figure S9.

Supplementary Notes 7: Additional experimental results on device 2

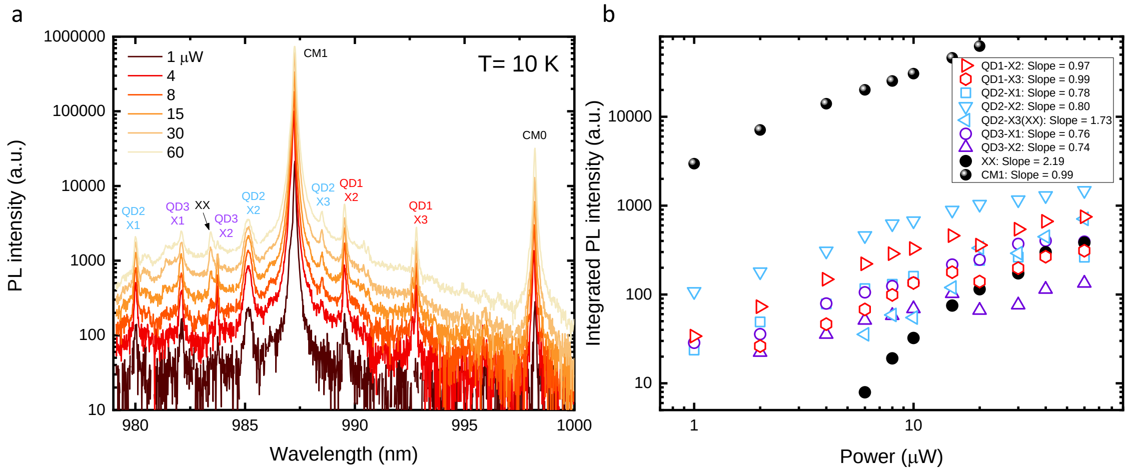

Figure S10 | Excitation power dependent PL measurement of device 2. a, Power-dependent PL spectrum of device 2. Black box: additional peaks are observed when power is increased. T = 10 K. **b,** Integrated PL intensity of QD exciton as a function of pump power. Linear fits to the data leads to slopes that are specified in the legend. λ_{exc} = 900 nm.

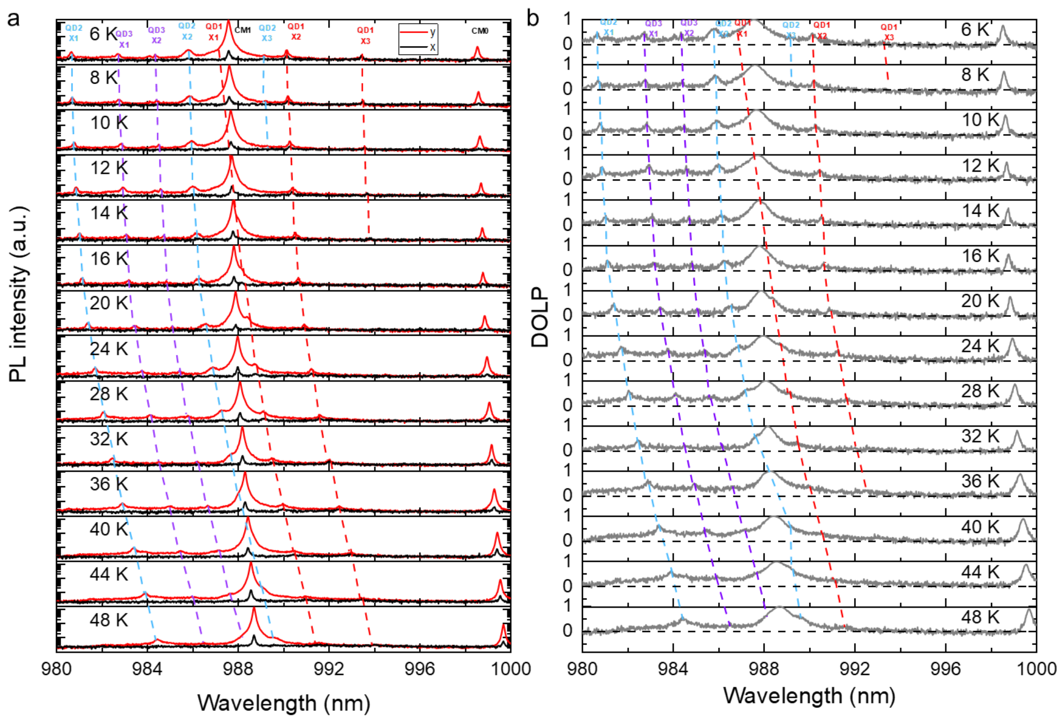

Figure S11 | Comprehensive temperature-dependent measurement data of device 2. a, Temperature-dependent polarization-resolved µPL of device 2. **b,** Temperature-dependent DOLP of device 1. Temperature varies from 6 K to 48 K. λ_{exc} = 900 nm.

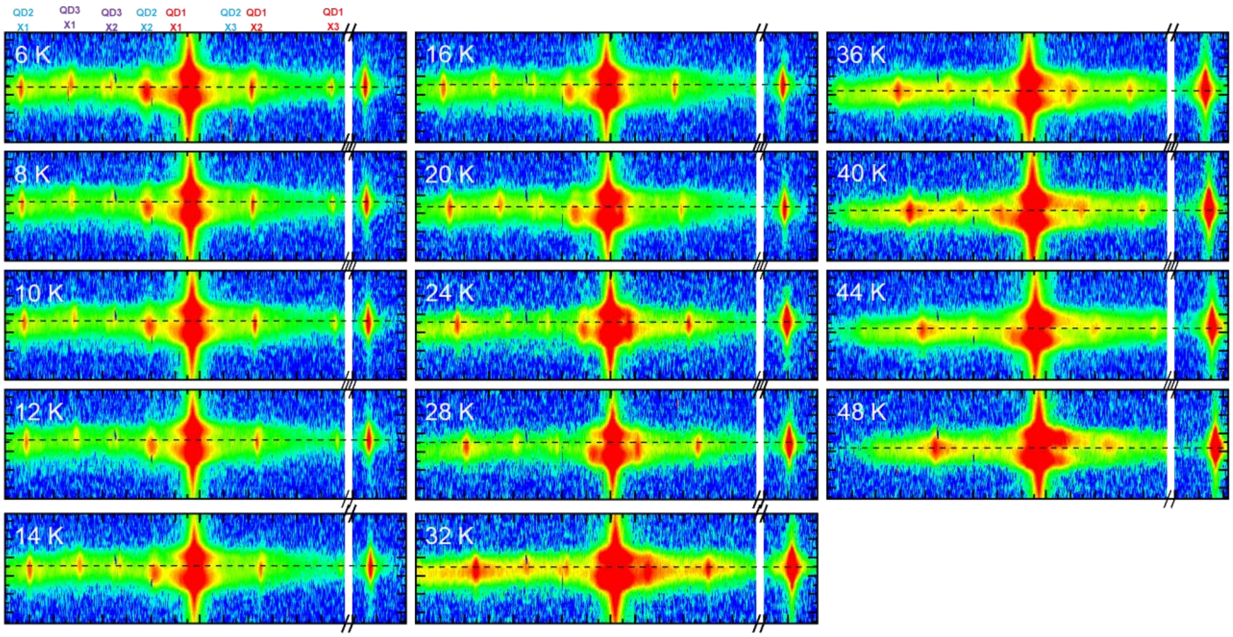

Figure S12 | CCD image of the temperature dependent position-wavelength map of QD excitons and CM1 for device 2. Temperature varies from 6 K to 48 K. λ_{exc} = 900 nm.

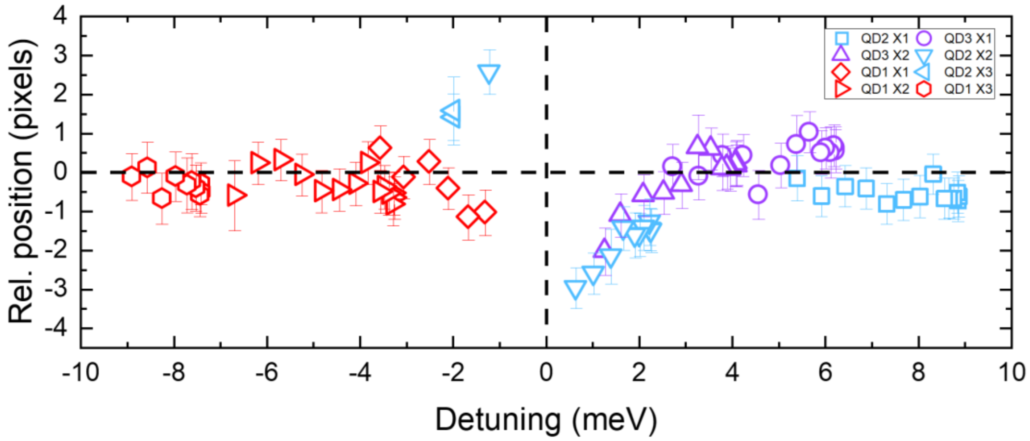

Figure S13 |The relative spatial position of excitons corresponding to QD1-3 with respective to the center of CM1 as a function of detuning. Mixed polarization is examined here.

Polarization-resolved PL spectra (including DOLP) and position-wavelength mapping of device 2 for the whole temperature range are shown in Figures S9 and S10. The relative spatial position of QD excitons with polarization not resolved are summarized in Figure S11.

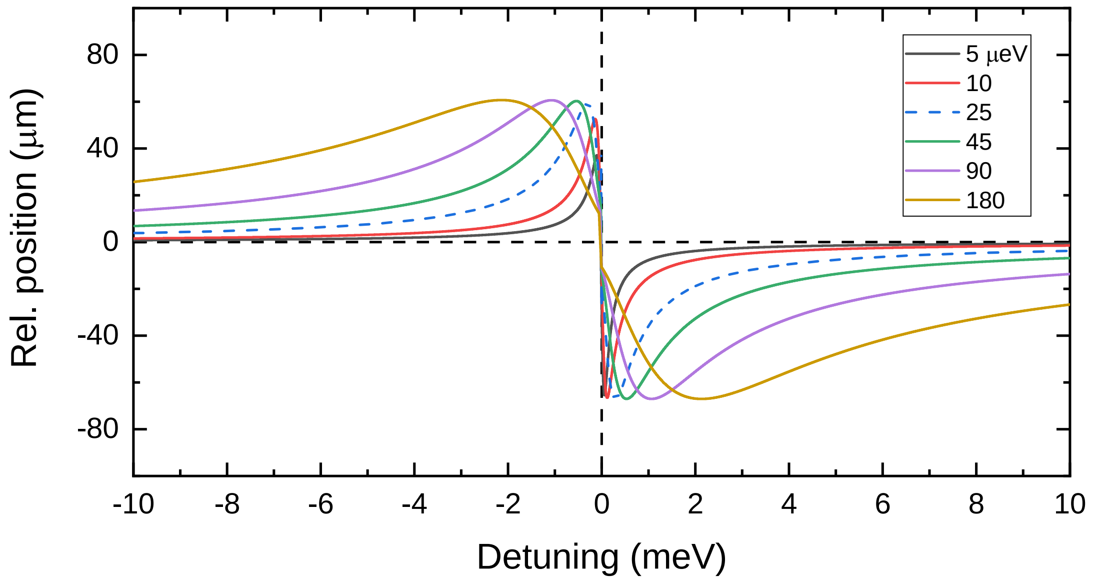

Figure S14 | W_{y21}/W_{y22} curves for device 2 in Figure 6c with coupling strength $g_2 = 5, 10, 25, 45, 90, 180$ μeV. κ = 100 μeV, γ_y = 0.7 μeV, χ_{21} = 0.9, χ_{22} = 0.05. The blue dashed curve represents $g_2 = \kappa/4 = 25$ μeV.

W_{y21}/W_{y22}curves for device 2 in Figure 6c with coupling strength $g_2 = 5, 10, 25, 45, 90, 180$ μeV are shown in the Figure S14. The spatial-repulsion feature of QD2 exciton, indicating the transition of QD2 emission between the two lobes in the CM1, exhibits a smooth transition from small g to large g value. It suggests that the spatial feature occurs in both weak and strong coupling regime, and the strong coupling is not a necessary condition. However, the detuning range where the spatial effect occurs increases when the coupling strength is large. It suggests that such quantum interference effect is more easily to be experimentally identified if the QD-cavity system operates in the strong coupling regime. Application wise, using our device for photon routing as proposed in Figure 7 prefers a system operating in the strong coupling regime with a large coupling strength.

Supplementary Notes 8: PL measurements of QD-CM0 interactions on device 3

CM0 is tuned around QD s-shell emission for device 3 (r = 36 nm). Figure S15 shows the PL scanning and the results are summarized as intensity bars on top of each PL peak. The spectrum at the bottom is measured when the laser spot is around the center of the cavity. Nine exciton lines are identified and associated to four QDs. Polarization-resolved PL spectra (including DOLP) and position-wavelength mapping of device 3 for the whole temperature range are shown in Figures S16 and S17.

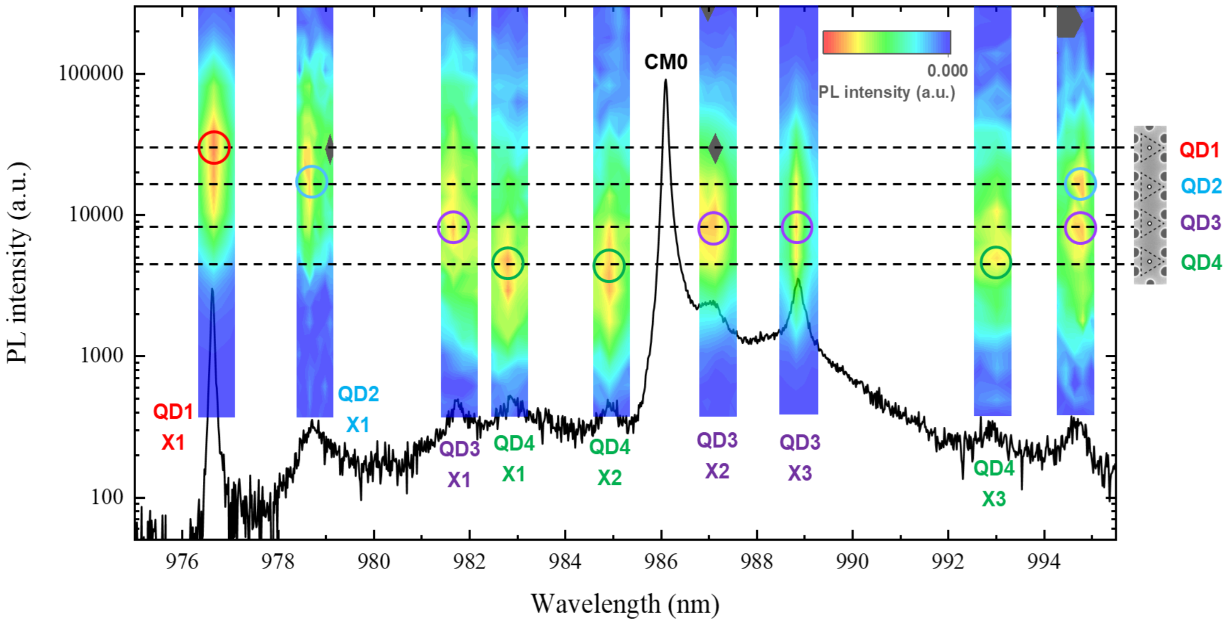

Figure S15 | Spatial scanning map of four-QD exciton emissions for device 3. The spectrum at the bottom is obtained around the center of the cavity. The brightness of different QD exciton lines is shown as bars where the vertical axis corresponds to the scanning steps.

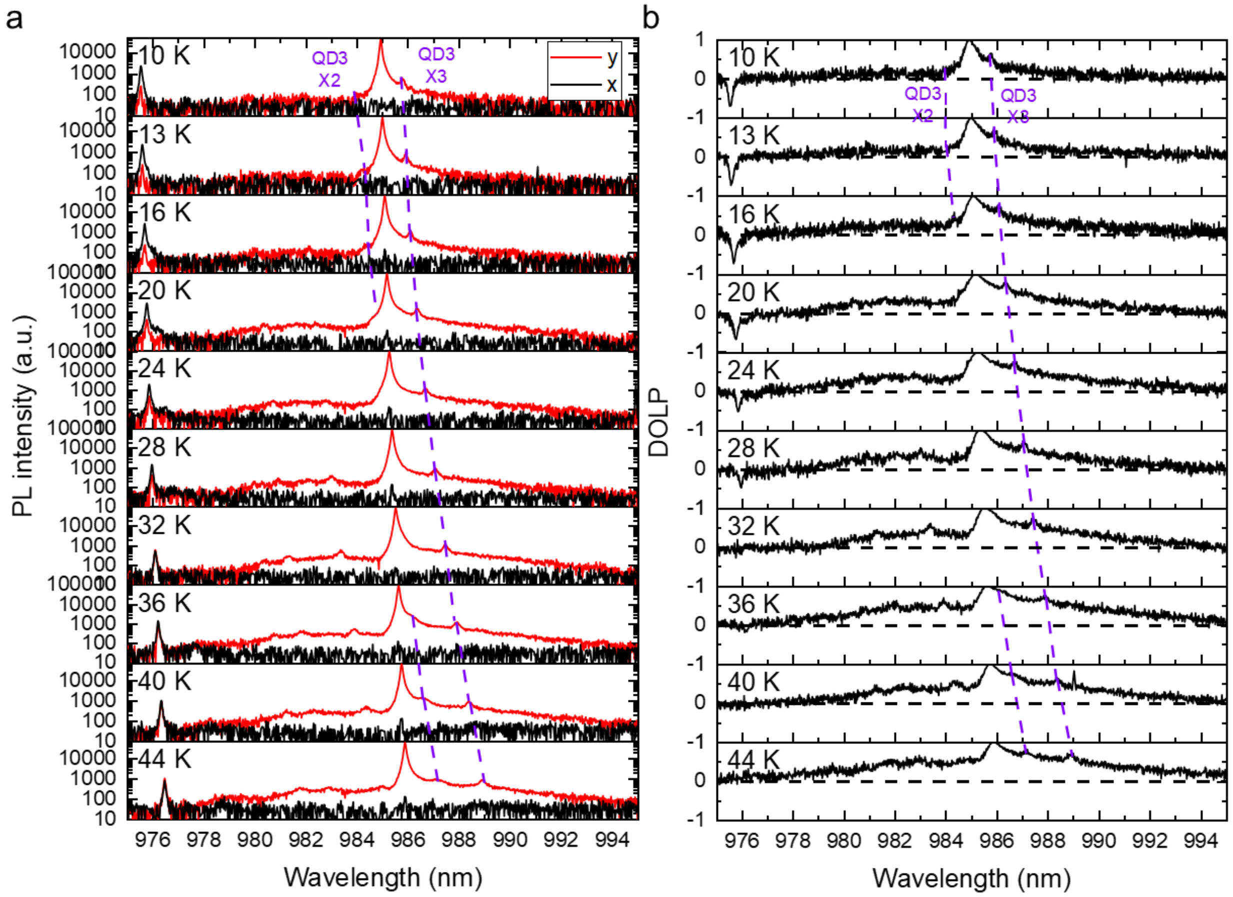

Figure S16 | Temperature-dependent measurements of device 3 for reference. a, Temperature-dependent polarization-resolved µPL of device 3. **b,** Temperature-dependent DOLP of device 1. Temperature varies from 10 K to 44 K. λ_{exc} = 900 nm.

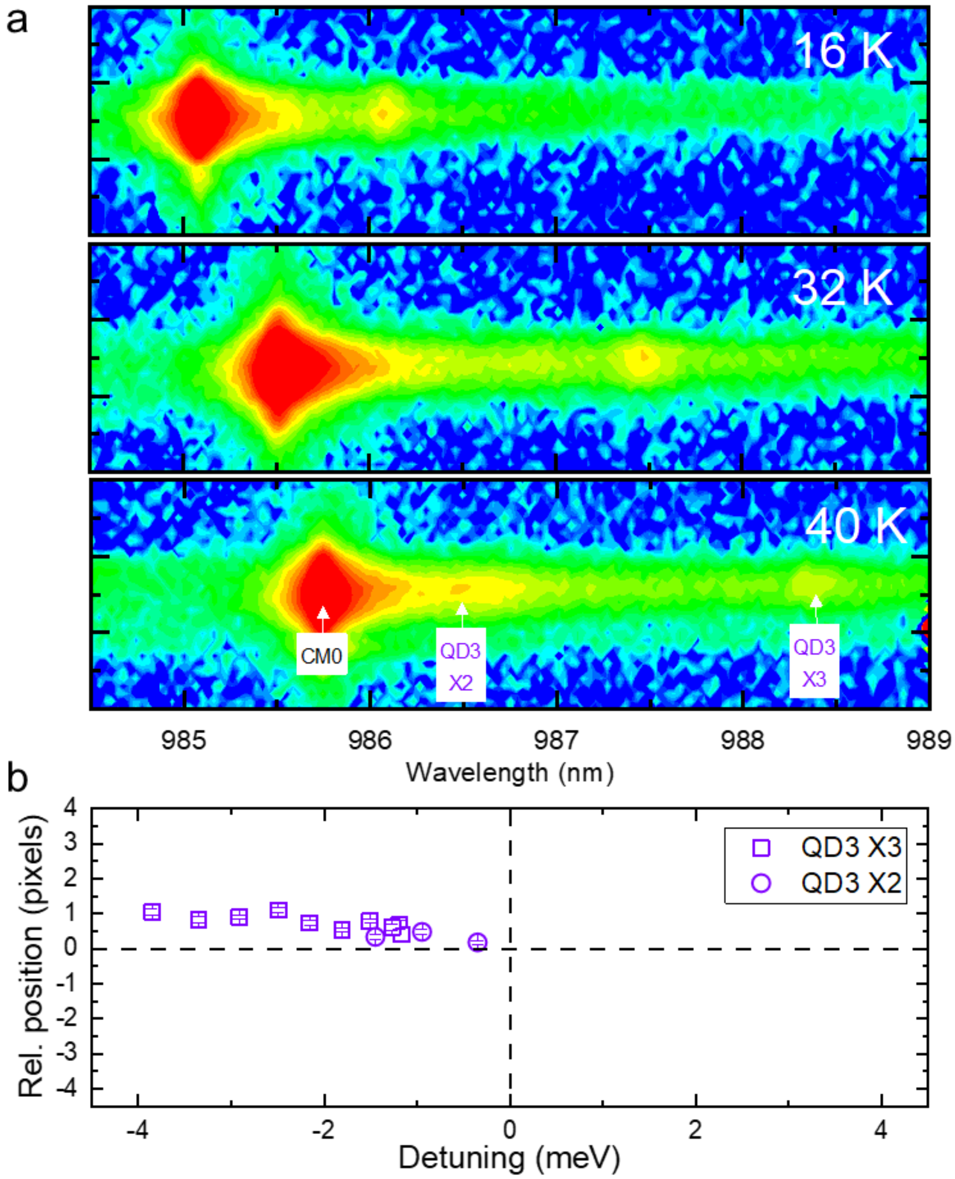

Figure S17 | Spatial- and spectrally-measurements of device 3 for reference. a, CCD image of the position-wavelength map of QD excitons and CM0 for device 3 at T = 16 K, 32 K, and 40 K. **b,** The relative spatial position of QD excitons QD3-X3 and QD3-X2 with respect to the center of CM0 as a function of detuning. Mixed polarization is examined here.

Supplementary Notes 9: Derivation of total QD emission rate into the y-polarized free space mode W_y

Consider the Hamiltonian of the QD-CM system coupled to the *y*-polarized free space modes as $\hat{\mathcal{H}} = \hat{\mathcal{H}}_0 + \hat{\mathcal{H}}_{int} + \hat{\mathcal{H}}_R$ [3, 4], where

$$\hat{\mathcal{H}}_0 = \hbar\omega_0\hat{\sigma}^\dagger\hat{\sigma} + \hbar\omega_c\hat{a}^\dagger\hat{a} + \hbar\sum_{\boldsymbol{k}}\omega_{\boldsymbol{k}}\,\hat{b}_{\boldsymbol{k}}^\dagger\hat{b}_{\boldsymbol{k}}, \tag{S1}$$

$$\hat{\mathcal{H}}_{int} = \hbar(g\hat{\sigma}^\dagger\hat{a} + \mathrm{H.c.}), \tag{S2}$$

$$\hat{\mathcal{H}}_R = \hbar\sum_{\boldsymbol{k}}(\xi_{\boldsymbol{k},y}\hat{a}^\dagger\hat{b}_{\boldsymbol{k}} + \mathrm{H.c.}) + \hbar\sum_{\boldsymbol{k}}(\eta_{\boldsymbol{k},y}\hat{\sigma}^\dagger\hat{b}_{\boldsymbol{k}} + \mathrm{H.c.}). \tag{S3}$$

Here ω_0 and ω_c are the QD transition energy and cavity mode resonance. $\hat{\sigma}$, $\hat{a}$, and $\hat{b}_{\boldsymbol{k}}$ are the annihilation operators of the QD exciton, CM, and free-space modes, respectively. $g = |g|e^{i\phi_g}$, $\xi_{\boldsymbol{k},y} = |\xi_{\boldsymbol{k},y}|e^{i\phi_\xi}$, and $\eta_{\boldsymbol{k},y} = |\eta_{\boldsymbol{k},y}|e^{i\phi_\eta}$ are the coupling strength of QD exciton with CM, CM with *y*-polarized free-space modes, and QD with *y*-polarized free-space modes, respectively.

For simplicity, consider the CM and free-space modes as Fock states with 0 or 1 photons and the QD exciton as two-level system with ground and excited state $|g\rangle$ and $|e\rangle$. The superposition wave function of the QD-CM system coupled to free space modes can be written as [3, 4]

$$|\psi(t)\rangle = a(t)e^{-i\omega_0 t}|e,0_c,0_{\boldsymbol{k}}\rangle + c(t)e^{-i\omega_c t}|g,1_c,0_{\boldsymbol{k}}\rangle + \sum_{\boldsymbol{k}} b_{\boldsymbol{k}}(t)\,e^{-i\omega_{\boldsymbol{k}} t}|g,0_c,1_{\boldsymbol{k}}\rangle. \tag{S4}$$

Here $|e,0_c,0_{\boldsymbol{k}}\rangle$, $|g,1_c,0_{\boldsymbol{k}}\rangle$, and $|g,0_c,1_{\boldsymbol{k}}\rangle$ are the Fock wave function corresponding to a single excitation in the system, a single photon in the CM, and a single photon in the free space mode, respectively. Evolution of the amplitude of three components can be obtained by solving the Schrodinger's equation using the Weisskopf-Wigner approximation [3, 4] as

$$\frac{da(t)}{dt} = -(ige^{i\phi_g} + \tilde{\chi}\frac{\sqrt{\gamma_y\kappa}}{2})c(t)e^{i\delta t} - \frac{\gamma_y}{2}a(t), \tag{S5}$$

$$\frac{dc(t)}{dt} = -(ige^{-i\phi_g} + \tilde{\chi}^*\frac{\sqrt{\gamma_y\kappa}}{2})a(t)e^{-i\delta t} - \frac{\gamma_y}{2}c(t), \tag{S6}$$

where γ and κ are the decay rates of QD exciton and CM. δ is the detuning of QD and CM. $\tilde{\chi} = \chi e^{-i\phi_{\xi\eta}}$ is the complexe overlap term of CM and QD exciton coupling to the common free space modes. The set of differential equations can be solved and the eigenenergies are [3, 4]

$$\gamma_{\pm} = -\frac{1}{2}\left(\frac{\kappa+\gamma_y}{2} - i\delta\right) \pm \frac{1}{2}\sqrt{\left(\frac{\kappa-\gamma_y}{2} - i\delta\right)^2 - 4(|g|^2 - i\chi|g|\sqrt{\gamma_y\kappa}cos\phi - \frac{\chi^2\gamma_y\kappa}{4})}. \quad \text{(S7)}$$

For our QD-CM system, $\kappa \gg \gamma$, then the total QD exciton emission rate into y-polarized free space mode is [3, 4]

$$W_y(\delta) = -2\mathrm{Re}(\gamma_+) = \frac{\kappa+\gamma_y}{2} - \mathrm{Re}[(\frac{\kappa-\gamma_y}{2} - i\delta)^2 - (2|g| - i\chi\sqrt{\gamma_y\kappa}e^{-i\phi}) \times (2|g| - i\chi\sqrt{\gamma_y\kappa}e^{i\phi})]^{1/2}. \quad \text{(S8)}$$

Supplementary References

[1] A. Tiranov, V. Angelopoulou, C. J. V. Diepen, B. Schrinski, O. A. D. Sandberg, Y. Wang, L. Midolo, S. Scholz, A. D. Wieck. A. Ludwig, A. S. Sørensen, and P. Lodahl, Collective super- and subradiant dynamics between distant optical quantum emitters. *Science* **379**, 389-393 (2023).

[2] J. Q. Grim, A. S. Bracker, M. Zalalutdinov, S. G. Carter, A. C. Kozen, M. Kim, C. S. Kim, J. T. Mlack, M. Yakes, B. Lee, and D. Gammon, Scalable in operando strain tuning in nanophotonic waveguides enabling three-quantum-dot superradiance. *Nat. Mater.* **18**, 963–969 (2019).

[3] A. Lyasota, C. Jarlov, M. Nyman, A. Miranda, M. Calic, B. Dwir, A. Rudra, A. Shevchenko and E. Kapon, Mode Interference Effect in Optical Emission of Quantum Dots in Photonic Crystal Cavities. *Phys Rev X* **12,** 021042 (2022).

[4] M. Yamaguchi, T. Asano, M. Fujita, and S. Noda, Theoretical Analysis of Light Emission from a Coupled System of a Photonic Nanocavity and a Quantum Dot. *Phys. Status Solidi C* **5**, 2828 (2008).
